# Tumor Endothelial Marker 8 Amplifies Canonical Wnt Signaling in Blood Vessels

**DOI:** 10.1371/journal.pone.0022334

**Published:** 2011-08-01

**Authors:** Kiran Verma, Jingsheng Gu, Erica Werner

**Affiliations:** 1 Department of Cell Biology, Emory University School of Medicine, Atlanta, Georgia, United States of America; 2 Department of Biochemistry, Emory University School of Medicine, Atlanta, Georgia, United States of America; University of Illinois at Chicago, United States of America

## Abstract

Tumor Endothelial Marker 8/Anthrax Toxin Receptor 1 (TEM8/ANTXR1) expression is induced in the vascular compartment of multiple tumors and therefore, is a candidate molecule to target tumor therapies. This cell surface molecule mediates anthrax toxin internalization, however, its physiological function in blood vessels remains largely unknown. We identified the chicken chorioallantoic membrane (CAM) as a model system to study the endogenous function of TEM8 in blood vessels as we found that TEM8 expression was induced transiently between day 10 and 12 of embryonic development, when the vascular tree is undergoing final development and growth. We used the cell-binding component of anthrax toxin, Protective Antigen (PA), to engage endogenous TEM8 receptors and evaluate the effects of PA-TEM8 complexes on vascular development. PA applied at the time of highest TEM8 expression reduced vascular density and disrupted hierarchical branching as revealed by quantitative morphometric analysis of the vascular tree after 48h. PA-dependent reduced branching phenotype was partially mimicked by Wnt3a application and ameliorated by the Wnt antagonist, Dikkopf-1. These results implicate TEM8 expression in endothelial cells in regulating the canonical Wnt signaling pathway at this day of CAM development. Consistent with this model, PA increased beta catenin levels acutely in CAM blood vessels in vivo and in TEM8 transfected primary human endothelial cells in vitro. TEM8 expression in Hek293 cells, which neither express endogenous PA-binding receptors nor Wnt ligands, stabilized beta catenin levels and amplified beta catenin-dependent transcriptional activity induced by Wnt3a. This agonistic function is supported by findings in the CAM, where the increase in TEM8 expression from day 10 to day 12 and PA application correlated with Axin 2 induction, a universal reporter gene for canonical Wnt signaling. We postulate that the developmentally controlled expression of TEM8 modulates endothelial cell response to canonical Wnt signaling to regulate vessel patterning and density.

## Materials and Methods

### Reagents and cell lines

Human recombinant VEGF165, recombinant mouse Wnt3a and DKK-1 (R&D Systems), DQ-PA and FITC-labeled PA (List Biological Laboratories). Hek293 cells (QB-293) were obtained from Qbiogene.

### Qualitative RT-PCR

Total RNA (1 µg) purified from CAM and liver extracted at the indicated day of development, was used to reverse transcribe mRNA with oligoT using AccuScript (Promega). PCR to detect gene expression was performed using 100 ng cDNA as a template and optimized at 40 cycles and annealing at 48°C. The bands were quantified by densitometry using ImageJ analysis software. The identity of all PCR products was confirmed by sequencing. The forward and reverse primer pairs (5′ to 3′) used were: TEM8 (XM_425758.2) tgagagggaggccaatcggtca and gcagcggcccttgtctcctg; CMG2 (XM_420539) gcagattgagaagcagggag and tgcatgactgcttcaacac; GAPDH (NM_204305) ggagtcaacggatttggcc and gtcacgctcctggaagatag; VE cadherin (NM_204227.1) atctcagacaacggcaatcc, and gaccgctcagatccttcttg; Axin-2 (NM_204491.1) tgccccgcggaggaatgag and ctctgacgcccgccgtaac.

### Eggs and CAM preparation

Fertilized Leghorn chicken eggs were obtained from a local provider and kept in a 37°C, 60% humidity incubator for 7 days. The CAM was dropped and the window sealed with stretchy tape (Scotchgard, 3M). At the day of the experiment, 3M filter paper squares with a 0.8 cm diameter hole were sterilized by soaking in 70% ethanol, air-dried in a sterile air cabinet, soaked in 2.5 mg/ml cortisone acetate in 95% ethanol to prevent an inflammatory response to the filter and placed on the CAM. Proteins and growth factors were diluted in 15 µL avian Ringer solution and delivered in the center of the hole. After 2 days, the filters were fixed *in ovo* with 4% paraformaldehyde in PBS and dissected. The filters showing surrounding blood vessels of normal appearance, were photographed using a Stemi SV6 stereomicroscope (Zeiss) equipped with a Digital Photo color camera DFC 500 (Leica).

### In situ hybridization

Filters were placed on the CAM at day 10 and fixed for 20 min *in ovo* at day 11 with 4%PFA. The filters were excised, bleached for 1 h with 0.3% H_2_O_2_ and treated with 5 µg/ml proteinase K in PBS for 5 min at room temperature.

Antisense (test) and sense (control) single-stranded RNA probes were generated using Riboprobe in vitro transcription kit (Promega), using as a template the PCR product cloned in pCII vector (Invitrogen). CAM were treated with 0.1 M Triethanolamine pH 7.5, and dehydrated in ethanol series (%50, %75, %95). After hydrated, they were pre-hybridized at 55°C for 1 hour in 0.3 M NaCl, 20 M Tris-HCl pH 8, 5 mM EDTA, 1x Denhardt's reagent, 1% Sarcosyl, 50% formamide 10% dextran sulfate, 0.25 mg/ml yeast tRNA (Sigma). Probes were denatured at 80°C for 5 minutes, chilled in ice and diluted in hybridization buffer. CAM were hybridized with their probes (200 µL each) for 16 to 20 hours at 55°C. To remove nonspecifically bound probes, CAM were washed in 2x SSC, 0.5x SSC at 56°C. Hybridization signals were visualized with antidigoxigenin antibody–alkaline phosphatase conjugates using 5-bromo-4-chloro-3-indoylphosphate and nitro-blue tetrazolium as chromogens (Boheringer Manheim).

### Morphometric analysis of Angiogenesis

For morphological analysis, the pictures were converted into binary images using ImageJ after converting to 8-bit grayscale, two-fold background subtraction with a rolling ball of 50 and 20 radius respectively and contrast/brightness adjustments as necessary. The binary images were analyzed using AngioQuant software, an automated image analysis tool developed to quantify in vitro angiogenesis assays by measuring number of tubules complexes, their size, length and number of branching points [1].

### TEM8 expression in HMEC

Primary Human Microvascular Endothelial cells were provided by Dr. Kowalczyk and cultured in EBM-2 (Lonza). A confluent monolayer was infected using a recombinant adenovirus to overexpress HA-tagged TEM8 or GFP (control) [40]. After 18h, the monolayer was incubated with or without 1 µg/ml PA for 3h and analyzed by western blot using anti active beta catenin (ABC, clone 8E7), HA tag, anti pan-beta catenin and tubulin antibodies (Sigma).

### Whole mount immunofluorescence

Filters were fixed *in ovo* with 4%PFA for 20 min. After excision, they were blocked for 2 h in 1% BSA, 0.1% Triton-X100 in PBS. Primary antibodies for FITC (Invitrogen), smooth muscle alpha actin and beta catenin (Sigma) or avß3 integrin (Millipore) were incubated overnight at 4°C. After washes in BSA-TX100, Alexa conjugated secondary antibodies were incubated at 1∶1000 dilution in BSA-TX100 overnight at 4°C. The filters were embedded in gelvatol for imaging using a confocal microscope equipped with 488 nm Argon and 568 nm HeNe lasers (LSM510; Carl Zeiss). Images were acquired at 2048x2048 pixel resolution, without gamma adjustment and at 8-bit depth at identical conditions for all samples in the same experiment. Brightness and contrast was adjusted to all images after assembling the figure panels.

### Nuclear/cytoplasmic ratio of beta catenin levels

To measure relative levels of beta catenin, a line was traced along the longitudinal axis of the cell nucleus on unprocessed LSM images of beta catenin staining using the line tool of Methamorph software. The intensity of each pixel in the line was recorded using the linescan measurement tool and compared to the pixel intensity when the line was placed on the cytosol area of lowest staining in the same cell. Thus, this ratio is independent of intercellular variability in background staining as well as variations in relative TEM8 and beta catenin expression levels.

### TCF/LEF Reporter Gene Assay

Wild-type and TEM8 expressing Hek293 cells were seeded on 35 mm dishes and transfected using Lipofectamine 2000 (Invitrogen) and 4 µg/ml of reporter plasmid (M50 Super 8x TOPFlash, obtained via Addgene from Dr. Randall Moon [2]). After 24 h, the transfected cells were seeded on 96 well plates and treated with or without recombinant mouse Wnt3a and/or PA at the indicated concentrations. Cells were lysed 18h later using Steady-Glo reporter substrate and lysis buffer (Promega) and luciferase activity was measured with a SynergyHT plate reader (BioTek). Cell number was corrected by protein concentration.

### Statistics

For statistical analyses, a non-paired student t test assuming equal variance was applied. A probability value of less than 0.05 was considered significant. The n values refer to replicates from separate animals or independently generated cell lines. When more than 2 samples were compared, a Student-Newman-Keuls Multiple Comparison test was applied.

## Introduction

TEM8 is an integrin-like membrane protein that is highly expressed in endothelial cells lining blood vessels in colon tumors [3]. Subsequent studies have shown that while this molecule is expressed at low levels in normal tissues, it is induced in the vascular compartment of multiple primary tumors and metastases, but not during normal vascularization occurring during wound healing or corpus luteum formation [4], [5]. This distinct expression pattern poises TEM8 as a candidate molecule to selectively target tumor-associated vasculature. In pre-clinical studies, fusion molecules between a receptor binding protein and a toxic enzymatic moiety, show potent anti-tumor and anti-vascular effects [6], [7]. The design, testing, and use of TEM8-directed therapies would benefit from further mechanistic knowledge about TEM8 function in endothelial cells and in blood vessels [8]. Furthermore, TEM8 function could reveal novel properties of tumor-associated blood vessels. However, the physiological function of TEM8 in the vascular compartment remains unknown.

TEM8 shares extensive structural and functional homology with Capillary Morphogenetic molecule 2 (CMG2), which is more widely expressed in the organism and is induced during in vitro capillary morphogenesis in collagen gels [9]. Both cell surface molecules bind the cell-binding component of the anthrax toxin, Protective Antigen (PA), which mediates toxin complex internalization and facilitates release of the enzymatic components, lethal and edema factors, into the host cell cytosol (reviewed in [10]). A potential role of anthrax toxin receptors in vascular physiology is further highlighted by the characteristic alteration of vascular function in anthrax pathophysiology. In fact, vascular damage can be replicated by injecting the tripartite holotoxin into the blood stream of animal models [11], [12]. The holotoxin also disrupts vascular morphogenesis in zebrafish and retinal development in mice [13], [14]. Moreover, Anthrax toxin binding to TEM8/CMG2 has been exploited in preclinical studies to deliver fusion proteins to tumors and tumor associated blood vessels [15]. In most cases, however, endothelial cells express both receptors, where they are involved in cell proliferation, migration and promoting angiogenesis in vitro and in vivo ([16], [17], [18], [19] and our unpublished results). These observations have provided a limited understanding of TEM8-specific function in the vasculature since toxin-mediated cell binding is the defining mechanism affecting the vasculature in the aforementioned studies.

We identified the chicken embryo respiratory organ, the chorioallantoic membrane, as a suitable model to study TEM8-specific mechanisms in vascular development and physiology, because TEM8 expression is transiently induced in the absence of detectable levels of CMG2. CAM development recapitulates several phylogenetically conserved mechanisms for blood vessel formation. New vessels form by sprouting angiogenesis from pre-existing vessels, by in situ differentiation of precursor cells (vasculogenesis), and by intussusceptive microvascular growth. These vascular morphogenetic processes are observed in normal and pathological blood vessel generation in mammals and are regulated by highly conserved mechanism and molecules [20]. The dynamic nature of the vascular plexus in the CAM is illustrated by its capacity to vascularize grafted organs, tumor tissue, and tumor cells, reproducing some of the processes occurring during early tumor-induced angiogenesis. Furthermore, as we show in the current work, the semi-planar arrangement of blood vessels developing in a regular hierarchical pattern, makes this system amenable for quantitative morphometric analysis of blood vessel formation.

We found that TEM8 is selectively and transiently induced in the CAM during a discrete developmental stage of vascular morphogenesis, correlating with intussusceptive angiogenesis. Consistent with a functional role of TEM8 receptors at this stage, TEM8 engagement by PA led to PA accumulation in endothelial cells. Moreover, PA-TEM8 complexes disrupted blood vessel morphogenesis, reducing the number of branching events and first order blood vessels. These phenotypes were partially mimicked by Wnt3a and were reverted by the Wnt antagonist, Dikkopf-1. We propose that developmentally controlled expression of TEM8 regulates vessel patterning and density by fine-tuning canonical Wnt signaling.

## Results

### TEM8 is induced in the vascular compartment during chick chorioallantoic membrane development

**Figure 1:**
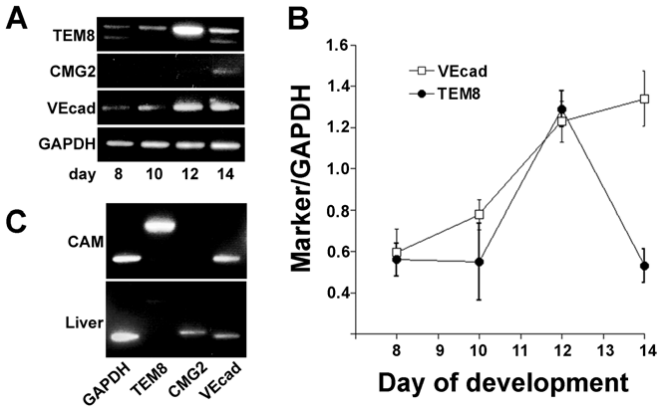
TEM8 expression is induced during chicken CAM development. **A** RT-PCR analysis of TEM8 expression over time. cDNA was prepared at the indicated day of development from total RNA isolated from CAM. Samples were standarized by GAPDH expression and analyzed with primers for TEM8, CMG2, VE-cadherin. **B** Average Ratio (+/−SEM) of TEM8 or VE-cadherin over GAPDH of 4–5 eggs collected at the indicated time of development. **C** Analysis of TEM8 and CMG2 expression by RT-PCR in CAM and liver at day 11 of development.

We hypothesized that if TEM8 plays specific roles in normal vascular development, then its expression should be restricted spatially to the vascular compartment of an organ and temporally to events modulated by this receptor. To test these predictions, we selected the chick chorioallantoic membrane, an organ possessing a hierarchical vascular development. CAM forms at day 5 of embryonic development when the chorionic ectoderm fuses with the allantoic endoderm. The membrane grows and expands until day 7 by endothelial cell proliferation and capillary sprout invasion of the mesenchyme to form a capillary plexus. Between days 8 and 12, blood vessels form by remodeling of the capillary plexus by intussusceptive angiogenesis until the CAM reaches a mature organization by day 12 [21]. To determine whether TEM8 is expressed in CAM blood vessels, we used RT-PCR to evaluate the expression of TEM8 and CMG2. We found that while TEM8 is expressed at low levels at early stages of CAM development and in the mature CAM (after day 12), there is a transient increase in expression that peaks between days 10 and 12 (Fig. 1A). In contrast, VE-cadherin expression, a marker for endothelial cells, showed a sustained increase during this period, consistent with continuous expansion of the vascular compartment (Fig. 1B). Interestingly, CMG2 mRNA expression was undetectable in the CAM until day 12 (Fig. 1A). To exclude differential sensitivity in receptor detection by RT-PCR, we analyzed TEM8 and CMG2 expression in the embryonic chicken liver at day 11. CMG2 was the predominant anthrax toxin receptor expressed in liver in contrast to TEM8, which was expressed at very low levels (Fig. 1C). These results show a differential expression of anthrax toxin receptors in the CAM and a brief temporal window when TEM8 expression is induced. These data suggest TEM8 participation in CAM morphogenesis in a temporally restricted developmental process.

**Figure 2:**
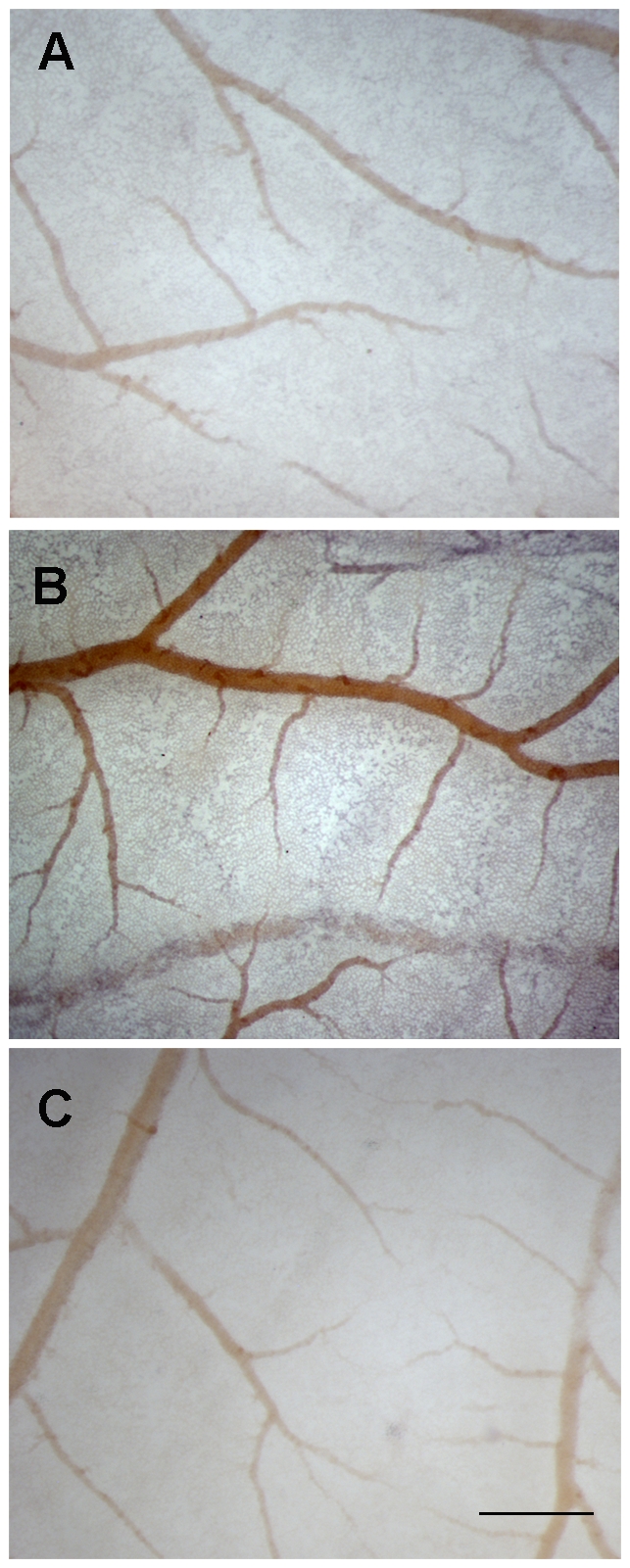
TEM8 is expressed in the vascular compartment of the CAM. In situ hybridization with sense **A**, anti-sense **B** or no probe **C** of whole mount 11 day CAM preparations. Scale bar =  500 µm.

**Figure 3:**
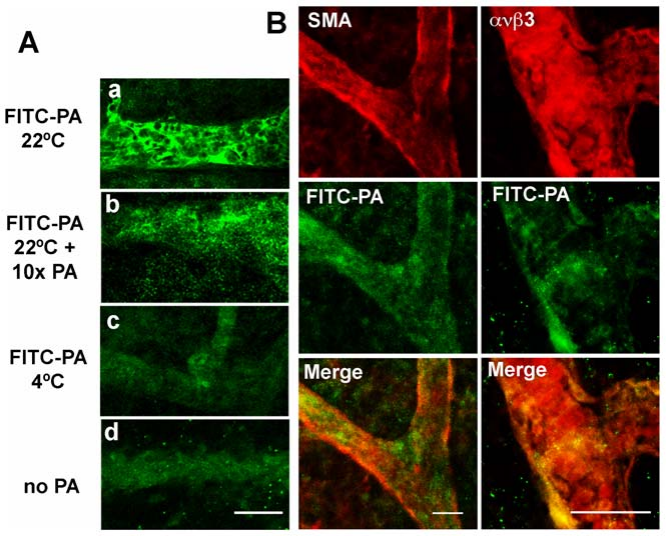
Labeled PA accumulates in CAM blood vessels. **A** Immunostaining for FITC label of day 11 CAM incubated for 2 h with 1 µg/ml FITC-PA at 22°C (a), with a 10 fold excess of unlabeled PA (b), at 4°C (c) or in the absence of PA (d). **B** Co-immunofluorescence for FITC and alpha smooth muscle actin marker for pericytes or avß3 marker for endothelial cells in day 11 CAM incubated for 2 h with 1 µg/ml FITC-PA at 22°C. Scale bar =  50 µm.

We asked next whether TEM8 expression is associated with the CAM vascular compartment. We used a two-pronged approach to determine the cellular compartment expressing TEM8. First, we generated antisense and sense cDNA probes labeled with digoxygenin-nucleotides to use in whole-mount *in situ* hybridization of CAM at day 11. Secondly, we used fluorescently labeled recombinant PA to identify the cellular compartment expressing anthrax toxin receptors. CAM hybridization with an anti-sense probe generated a signal specifically associated to blood vessels and the capillary plexus (Fig. 2B). The staining was heterogeneous, but continuous throughout the small caliber vascular compartment. In contrast, a sense probe (Fig. 2A) or no probe (Fig. 2C) produced negligible signal even though samples were processed under identical conditions. Next, we used FITC labeled recombinant PA to identify PA-binding structures or cells in the CAM. FITC-PA was detected by indirect immunofluorescence using an anti-FITC antibody. We incubated day 11 CAM with FITC-PA in vitro for 2h at 22°C in order to allow for intracellular accumulation of the labeled ligand due to recycling activity of the receptor [22]. As shown in Fig. 3, incubation at 22°C increases dramatically the sensitivity of the detection when compared to incubation at 4°C, an observation consistent with receptor-mediated endocytosis. FITC-PA binding was specific, because a 10-fold excess of non-labeled PA reduced the signal. We attribute incomplete competition by cold ligand to the mechanism for PA internalization, which requires the oligomerization of seven PA molecules [10]. FITC-PA was concentrated in fully formed blood vessels, in cells with a cobblestone appearance characteristic of endothelial cells. Co-staining with antibodies for cell-specific markers showed that PA accumulated mostly in avß3 positive cells, a marker previously shown to identify endothelial cells in the chicken CAM (Fig. 3B) [23]. In contrast, no co-localization was observed with anti smooth muscle actin, a marker for pericytes, which do not cover blood vessels completely at this day of CAM development [24]. In situ hybridization and immunodetection of FITC-PA point to small blood vessels and the capillary plexus as the most prominent structures expressing TEM8 at day 11 of CAM development. These results indicate that endothelial cells express functional TEM8 receptors.

### TEM8 receptor engagement with the ligand Protective Antigen (PA) reduces branching morphogenesis and blood vessel density, similar to canonical Wnt pathway activation

**Figure 4:**
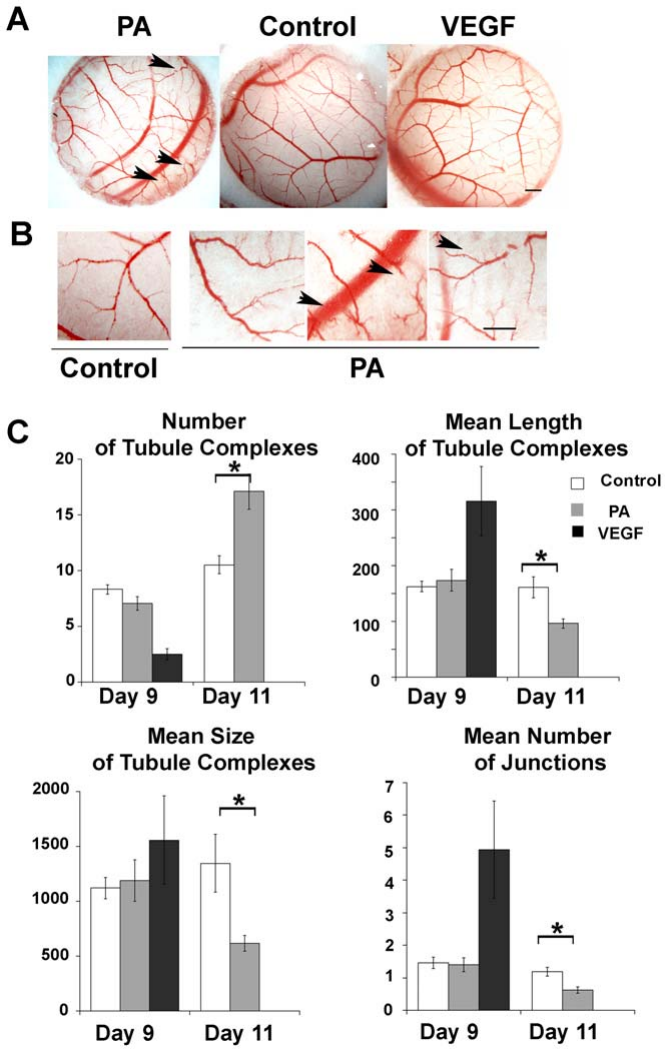
Protective Antigen disrupts blood vessel patterning in the CAM. **A** Images of filters on CAM at day 13, after treatment at day 11 with 1 µg PA, without agent or at day 9 with 100ng VEGF. Morphometric analysis of these filters with AngioQuant yields average values shown in (c). Scale bar = 1 mm. **B** Selected areas from control and PA-treated filters in (A) magnified to show details. Scale bar =  500 µm. Arrowheads point to a vessel discontinuity. **C** Average +/− SEM of the indicated AngioQuant parameter after analysis at day 13 from eggs treated or not at day 9 (n = 6) or day 11 (n = 10) with 1 µg of PA, or with 100 ng VEGF at day 9 (n = 6). * = p<0.05.

TEM8 expression analysis (Fig. 1) indicates that TEM8 is the major anthrax toxin receptor expressed in the CAM. Thus, PA can be used as a tool to either engage TEM8 activity or disrupt normal function by inducing receptor internalization and degradation [22], [25]. We took advantage of the accessibility of the CAM capillary plexus to exogenous agents to test whether PA binding to TEM8 modified CAM vasculogenesis or blood vessel function. We performed these experiments at day 11, 24 hours before the peak of TEM8 expression. PA application at day 11 decreased the caliber of blood vessels after 48 hours, which exhibited a pattern of reduced complexity and hierarchical branching (Fig. 4A). PA induced frequent blood vessel discontinuities (arrowheads) and disappearance of first order pre-capillary vessels (averaging 30 µm diameter), which is reflected in a reduced number of branches (Fig. 4B)[26]. In contrast, when 100 ng VEGF165 was applied on the CAM for 48 h, the size of complexes and number of branches increased without changes in their patterning.

The planar and hierarchical arrangement adopted by the newly formed CAM blood vessels is amenable to morphometric analysis applying strategies and parameters used to describe endothelial cell tubule formation *in vitro*. Therefore, we used AngioQuant software to quantitatively describe PA-induced changes in vessel branching, growth in length and diameter. To exclude effects unrelated to receptor-ligand interactions, we evaluated the effects of PA applied on CAM at day 9, when TEM8 is expressed at low levels. The effects of PA applied at day 11 were also compared to the effects of VEGF, a pro-angiogenic agent known to induce blood vessel growth and branching [27], [28]. The number of tubule complexes increased significantly from 100.5±7.9 to 170.9±15.9 (average ± SEM) when PA was applied at day 11, reflecting the increase in vessel discontinuities. In contrast, VEGF reduced the number of complexes per area from 83.3±4.2 to 25.2±4.9 (average ± SEM), by promoting a robust growth and expansion of complexes already existing at the beginning of VEGF treatment. The reduced complexity and number of primary branches in response to PA was reflected in a reduction in the total length of tubule complexes and the mean number of junctions from 161±19 to 96.4±8.3 and from 1.19±0.13 to 0.6±0.1, respectively. PA also reduced the mean size of complexes from 1346.8±264.8 to 615.2±72.5, a parameter describing both length and thickness of the blood vessels. No significant differences were observed when PA was applied at day 9, excluding effects by noxious contaminants present in recombinant PA and highlighting a dependence on TEM8 expression. Moreover, the effects of PA were abrogated by PA denaturation at 70°C for 15 min before application on the CAM and were transient, as branching and patterning recovered by day 14 (not shown). These PA-induced CAM vascular phenotypes are consistent with PA-TEM8 complexes preventing the formation of new branches or destabilization of the newly formed pre-capillary vessels.

**Figure 5:**
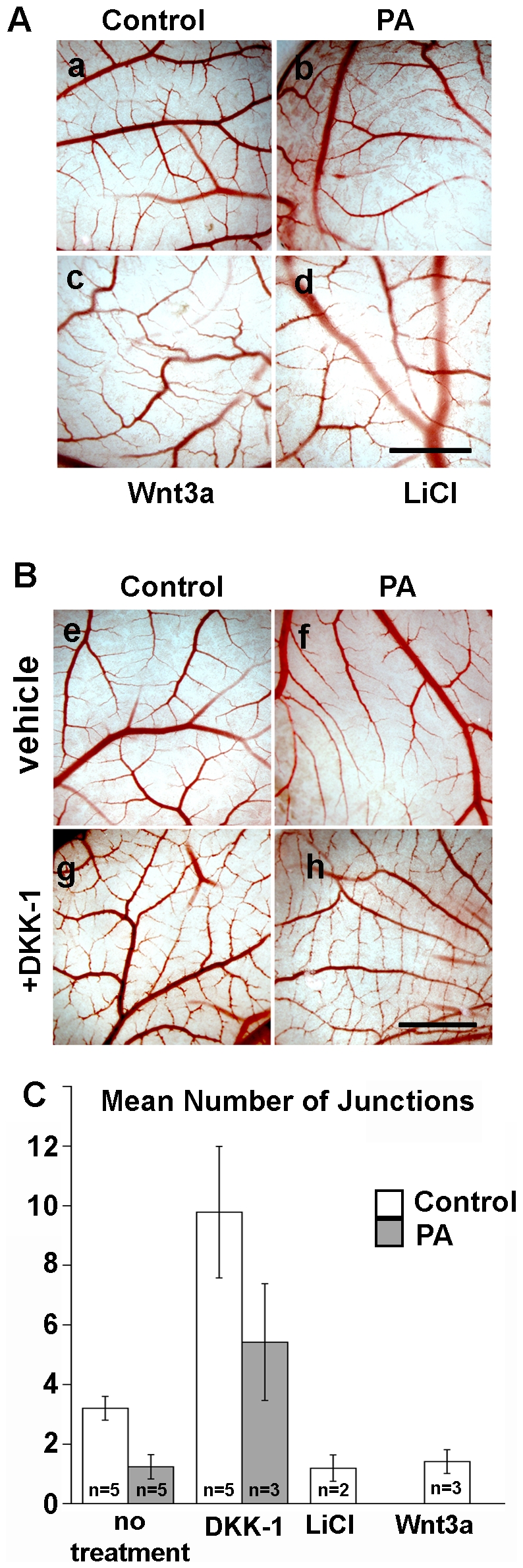
Morphological effects of PA are phenocopied or ameliorated by regulators of the canonical Wnt pathway. Day 11 CAMs were incubated with agonist of the canonical pathway, wnt3a or LiCl **(A)** or an antagonist DKK-1 **(B)** avian ringer solution (a,e), 1 µg PA (b,f,h), 200 ng DKK-1 (g,h), 100 ng mWnt3a (c) or 5 mM LiCl (d) and photographed after two days, at day 13 (Scale bar =  500 µm). **C** Quantitative analysis of the mean number of branches per blood vessel complexes in the indicated number of CAMs using AngioQuant. Error bars represent standard error.

The phenotypic effects of PA on CAM blood vessels are consistent with inhibition of branching events and/or reduced stability of the blood vessels formed at day 11 and 12. Members of the Wnt family have been implicated in blood vessel morphogenesis, remodeling and stabilization ([29], [30] and reviewed in [31]). Particularly, forced expression of beta catenin gain of function mutant in endothelial cells leads to a strikingly similar phenotype (see figure 2 in [32]). Wnt ligands bind to Frizzled receptors and LRP5/6 co-receptors. Ligand binding to receptors induces beta catenin stabilization by inhibiting GSK-3-dependent phosphorylation and thereby preventing beta-catenin targeting for proteasomal degradation. Dephosphorylated beta catenin translocates to the nucleus and relieves T-cell factor/lymphoid enhancer factor (TCF/LCF) repression of gene transcription [33]. Furthermore, additional reports link TEM8 to Wnt signaling: TEM8 interacts with LRP6, a co-receptor for Wnt ligands, and causes beta catenin stabilization in a cell line model system [34], [35]. The phenotypic similarities between PA-TEM8 effects in CAM and forced beta catenin signaling in endothelial cells in vivo, lead us to test the hypothesis that PA and/or TEM8 expression promotes Wnt beta-catenin signaling in the chicken CAM. We tested first whether exogenous Wnt application or pharmacological upregulation of the pathway would trigger a vascular branching phenotype similar to PA. Day 11 CAM was exposed to recombinant mouse Wnt3a, which triggers the activation of the canonical pathway, or to LiCl, which mimics Wnt pathway activation by inhibition of the GSK-3ß kinase. Similar to PA, Wnt3a or the Wnt-mimic LiCl, phenocopied the PA-induced branching pattern by reducing branching events (Fig. 5A compare c & d to b and quantification in Fig. 5C). Conversely, application of recombinant DKK-1 protein, an antagonist of endogenous Wnt binding to receptors [36], increased the number of branches per vessel in both control and PA treated CAMs (shown in Fig. 5B and quantified in Fig. 5C), indicating that endogenous Wnt controls the number of branching events during normal vasculogenesis. These results suggest that PA up-regulates canonical Wnt signaling resulting in a reduction in branch formation.

### Increased TEM8 expression and PA stabilize beta catenin pools in endothelial cells *in vitro* and *in vivo*

**Figure 6:**
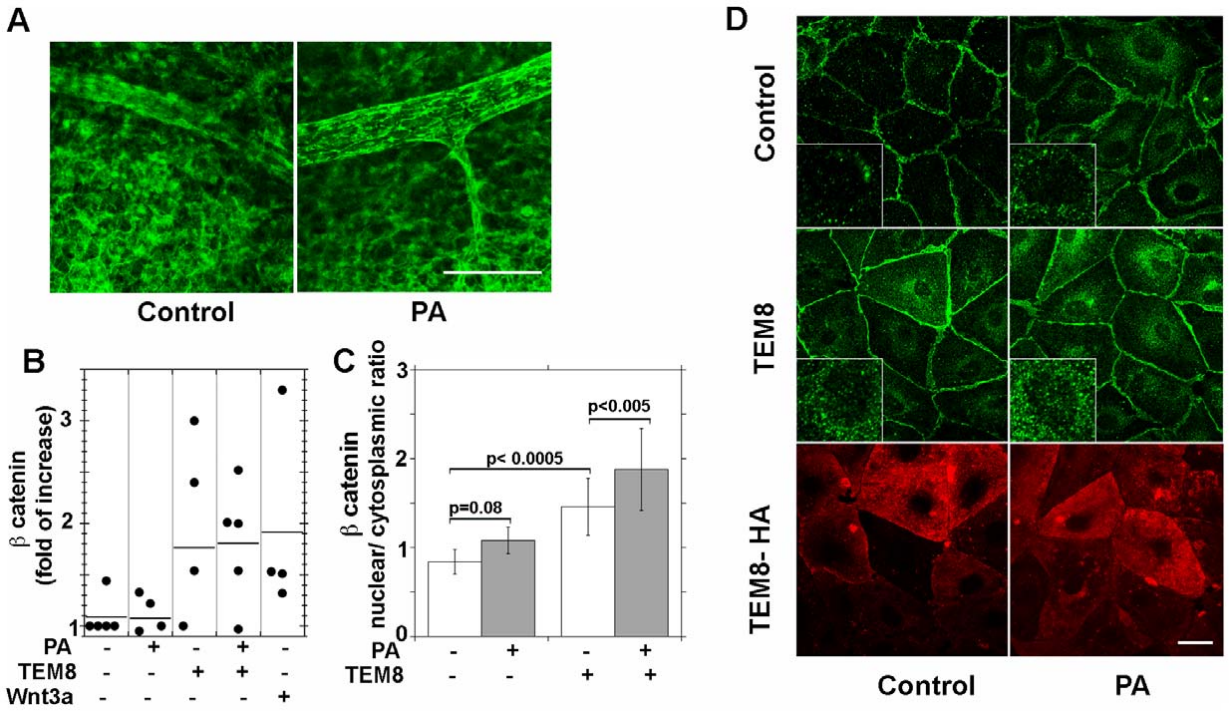
Beta catenin levels and distribution in response to TEM8 and PA in endothelial cells. **A** Beta catenin immunostaining of day 11 CAM after in vitro incubation for 3 h at 37°C in the absence or presence of 1 µg/ml PA in DME. Scale bar =  50 µm. **B** Beta catenin levels detected by western blot in lysates prepared from GFP- or TEM8-recombinant adenovirus infected HMEC treated with 100 ng/ml mWnt3a or 1 µg/ml PA for 3 h. The intensity of the bands was corrected by tubulin levels and are relative to untreated control cells (n = 4). **C** Relative beta catenin localization expressed as nuclear/cytoplasmic ratio (see materials and methods) in control or TEM8- recombinant adenovirus infected HMEC, treated or not with 1 µg/ml PA for 3 h. The data was collected in 20 cells from 2 independent experiments. **D** Immunoflurescence staining for beta catenin distribution in control and TEM8 expressing HMEC used to generate data shown in C. Size bar =  20 µm.

A hallmark for canonical Wnt signaling is the stabilization of beta catenin intracellular pools. We predicted that if TEM8 up-regulates Wnt signaling, then PA should stabilize beta catenin levels in TEM8 expressing endothelial cells. We tested this prediction both *in vivo* and *in vitro* using day 11 CAM and primary cultured human endothelial cells. CAM were excised at day 11 and incubated for 3h in the absence or presence of PA at 37°C followed by beta catenin staining. Beta catenin staining in untreated CAMs was faint and distributed in an inconspicuous pattern (Fig. 6A). Upon PA treatment, beta catenin overall staining intensity increased and localized to cell-cell junctions, most notable in first order vessels. The effects of PA *in vivo* were recapitulated *in vitro,* in primary cultured human microvascular endothelial cells. PA addition to endothelial cells expressing recombinant TEM8 induced significant beta catenin accumulation as determined by immunoblot (Fig. 6B) and at cell-cell junctions as visualized by immunofluorescence (Fig. 6D). The magnitude of beta catenin accumulation was comparable to the addition of recombinant Wnt3a to non-infected cells (Fig. 6B). Albeit not statistically significant, recombinant TEM8 expression alone was sufficient to increase beta catenin levels. We attribute the high variability in beta catenin levels to the presence of endogenous Wnt ligands, which are produced by endothelial cells in culture [37] and the low sensitivity of the assay for cells with abundant intercellular junctions [38]. Analysis for beta catenin translocation to the nucleus in individual cells, revealed that TEM8 overexpression and PA addition promoted beta catenin translocation to the nucleus as revealed by an increased nucleo-cytoplasmic ratio of beta catenin levels (Fig. 6C, and inserts in 6D). We sought further evidence for beta catenin activation by using an antibody specifically recognizing the dephosphorylated form of beta catenin (anti Activated Beta Catenin) [39]. This antibody did not yield a signal by immunofluoresence (not shown), but detected a small pool of activated molecules in Wnt3a treated endothelial cells and in PA treated TEM8-overexpressing endothelial cells (Fig. S1). These results indicate that TEM8 overexpression and PA binding to TEM8 in endothelial cells induces an increase in beta catenin total levels, at cell junctions and in the nucleus, and a pool of beta catenin competent to induce gene transcription.

### TEM8 and its ligand PA positively regulate Wnt induced gene transcription

**Figure 7:**
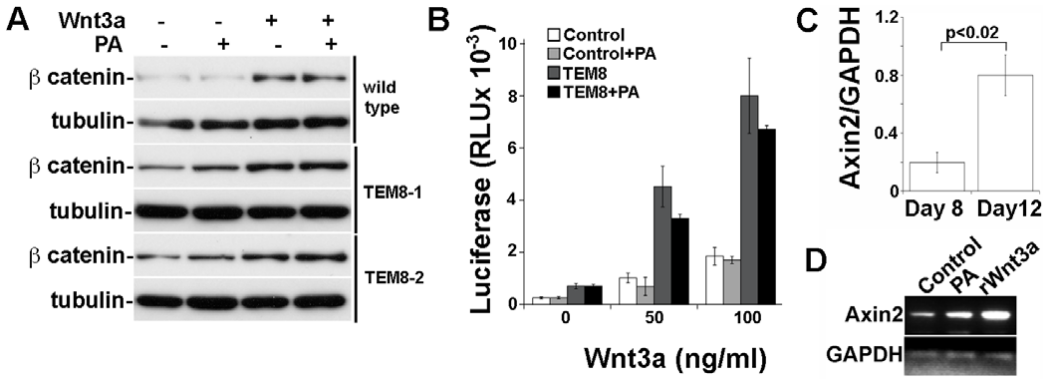
TEM8 increases beta catenin dependent transcriptional activity in vitro and in vivo. **A** Beta catenin levels detected by western blot in control and two independently generated TEM8-expressing HEK293 cell lines treated without or with 100 ng mWnt3a or 1 µg/ml PA for 3 h at 37°C. **B** Luciferase expression driven by a beta catenin responsive promoter (MF50 Super 8x TOPFlash) induced in control and TEM8 expressing HEK293 cells by increasing concentrations of Wnt3a in the absence or presence of 1 µg/ml PA for 18 h. This is one representative experiment of 3. Error bars are STDEV. **C** RT-PCR analysis for endogenous axin-2 expression in CAM at days 8 and 12 of development (+/− SEM, n = 3) **D** RT-PCR analysis of endogenous axin-2 expression in CAM at day 12 after a 16h treatment with vehicle (control), 1 µg of PA or 100 ng mWnt3a.

An increased nucleo-cytoplasmic ratio of beta catenin levels by TEM8-PA receptor-ligand complexes predicts a transcriptional induction of canonical Wnt-responsive genes. We tested this hypothesis by reconstituting TEM8- and PA-dependent beta catenin phenotypes in Hek293 cells. This cell line does not express endogenous anthrax toxin receptors and is responsive to Wnt ligands activating the canonical pathway [38], [40]. TEM8 expression was sufficient to increase basal and Wnt3a-induced levels of beta catenin as determined by immunoblot in at least two independently generated TEM8-expressing cell lines (Fig. 7A). TEM8 conferred sensitivity to PA, which increased beta catenin levels even further. We measured Wnt-dependent transcriptional activation with a reporter plasmid containing multiple TCF binding sites driving luciferase transcription (M50Super8xTOP Flash). Wnt3a was sufficient to induce luciferase expression in wild type cells (Fig. 7B) and TEM8 expression, although was not sufficient to promote gene transcription by itself, amplified the transcriptional response to Wnt3a, further increasing luciferase expression from 14.1±4.6-fold in control cells to 32.4±5.7 SEM in TEM8 expressing cells (Fig. 7B, average ± SEM). Surprisingly, PA equally reduced Hek293 cell response to Wnt3a irrespective of whether these cells expressed or not TEM8 (to 73.6±8.2% TEM-8 expressing cells and to 80.3±3.2% in control cells, n = 4). This TEM8-independent effect was also independent of PA concentration (not shown). Thus, we conclude that this is a receptor-independent effect of PA in this cell type. We attribute the lack of effect of PA on TCF transcriptional activation in Hek293 cells to a cell specific response.

Positive modulation of Wnt transcriptional response by TEM8 in Hek293 cells predicts changes in the induction of Wnt-responsive genes in the CAM associated to TEM8 expression and, possibly, to PA application. To study Wnt-dependent gene expression *in vivo,* the analyzed the mRNA levels by RT-PCR of a universal reporter gene for canonical Wnt pathway activity, Axin-2 [41]. Analysis of the same samples used in Fig. 1, showed that axin-2 expression increased from day 8 to day 12 of CAM development (Fig. 7C). In contrast to Hek293 cells, PA or Wnt3 application on CAM at day 10, increased Axin 2 expression as well (Fig. 7D). Altogether, these results support the notion that TEM8 is a positive regulator of Wnt signaling and that PA binding promotes this activity in endothelial cells in vitro and in the CAM in vivo.

## Discussion

We have defined a role for TEM8 in blood vessel morphogenesis acting as a modulator of Wnt-dependent vascular morphogenetic events. The highly stereotypic vascular development both in time and spatial organization of the chicken CAM permitted quantitative morphometric analysis of blood vessels in a nearly bidimentional organ. In situ hybridization and functional staining with labeled PA, indicate that in this organ, TEM8 is expressed in endothelial cells in a narrow window of time that correlates with organ vascularization by intussusceptive angiogenesis. At this precise developmental time, TEM8 engagement with its ligand PA alters blood vessel patterning and density affecting the hierarchical architecture of the vascular tree reducing vessel branching. These phenotypes are consistent with Wnt pathway activation.

TEM8 expression is up-regulated in CAM blood vessels between days 10 and 12 of embryonic development. Temporal expression of TEM8 coincides with a peak of intussusceptive angiogenesis. In contrast to sprouting angiogenesis, intussusceptive angiogenesis occurs by endothelial cell thinning and vessel splitting without involving proliferation, cell matrix degradation and cell migration [42]. This mechanism for vessel generation has been detected in lung, kidney, and mammary gland development by morphological criteria, because no biochemical markers have been identified so far [43], [44]. Intussusceptive angiogenesis is also found in some tumors and increases during tumor revascularization after radiation or anti-angiogenic therapy [45], [46], [47], and thus, is proposed as an alternative mechanism for blood vessel formation in tumor recurrences after therapy. An attractive possibility is that TEM8 may play a role in normal and/or pathological intussusceptive angiogenesis and may serve as a marker. However, further studies are required to test whether TEM8 function is directly linked to Intussusceptive angiogenesis.

Contrary to studies in endothelial cells in culture and in adult tissues, where CMG2 seems to be expressed and have a predominant role, we found that TEM8 is the predominant PA receptor in the CAM. The selective expression of TEM8 is supported by our RT-PCR experiments, which detected CMG2 expression in chick liver but not in the CAM at the time of the experiments. Furthermore, the temporal course of TEM8 expression correlated with a time-restricted CAM response to PA application. PA affected blood vessel patterning when applied at the peak of TEM8 expression, but not when TEM8 was expressed at low levels at day 9 (Fig. 4) or after day 12 (not shown). These results not only point to a role for TEM8 expression, but also support our conclusion that the effects induced by PA can be unequivocally attributed to TEM8. Coincident with TEM8 mRNA peak expression, we observed that fluorescently labeled PA accumulated in endothelial cells by a receptor-mediated mechanism, supporting the expression of functional receptors in this blood vessel compartment. PA applied to the CAM induced changes in blood vessel branching hierarchy and density with a reduction in primary vessels.

A reduced number in primary vessels and branching could result from deficiencies in formation, stabilization of the newly formed vessel or deregulated pruning. These processes would preserve early hierarchical patterning and produce a phenotype of apparent delayed or arrested development, while the observed phenotype is consistent with growth following altered spatial cues. Vascular density during blood vessel development is controlled by the cooperative activity of temporally and spatially regulated levels of Wnt and Notch to control tip cell activity and branch stability. The PA-induced changes in vessel patterning that we observed are strikingly similar to those resulting after expressing gain-of-function beta catenin targeted to endothelial cells during mouse development [32]. Paradoxically, a branching-deficient phenotype results from beta catenin-induced transcription of a ligand for Notch signaling, DLL4 [32]. DLL4 inhibits tip cell activity and thereby blocks blood vessel branch development [48], [49]. Thus, super activation of beta catenin by PA binding to TEM8 could lead to reduced branching by similar mechanism acting at the tip cell. Further experiments are needed to test this mechanism.

We propose that TEM8 modulates canonical Wnt signaling during normal vascular development in the CAM and PA functions as an agonist by engaging TEM8, further increasing beta catenin activation. This model is supported by the following findings: first, PA-induced morphological changes in the vascularization pattern are replicated by Wnt3a incubation or by treatment with LiCl, a non-selective Wnt pathway agonist. Second, PA induced alterations were attenuated when Wnt signaling was antagonized with DKK-1, which prevents endogenous Wnt from forming a complex with Frizzled and LRP5/6 [36]. DKK-1 also affected normal morphogenesis, increasing the number of branching events, suggesting that canonical signaling is necessary to determine branching events and blood vessel density. Hyper branching phenotypes are observed as a result of disruptive mutations in several components of the Wnt and Notch pathways, including beta catenin loss-of-function, Notch deficiency, and DLL4 Notch ligand inhibition [32], [50]. Thus, DKK-1 effects on normal CAM and on the PA-induced phenotype indicate that the canonical Wnt pathway becomes activated and regulates vascular density at this stage of vascular development. This model of TEM8-PA mediated Wnt signaling modulation is further supported by PA induced axin-2 expression. Third, PA affected a component of the canonical signaling pathway, inducing beta catenin stabilization in endothelial cells *in vivo* and *in vitro* as indicated by increased beta catenin staining at endothelial cell junctions in the CAM in vivo, in HMEC in vitro, and by western blot detection of protein levels. Although increased beta catenin levels at junctions could mediate adhesive functions, cadherins bind activated forms of beta catenin and this pool is competent for transcriptional activation [38], [51]. A role for this larger pool of beta catenin in gene transcription is supported by our findings of increased axin-2 expression in CAM correlating with TEM8 expression at day 12, with axin-2 induction after PA or Wnt3a treatment and with TEM8 dependent amplification of Wnt3a-induced transcription in Hek 293 cells.

Previous studies have reported a direct interaction of TEM8 with LRP6 and regulation of LRP6 levels by TEM8 when expressed in cell lines [34], [35]. However, this interaction was subsequently shown to be irrelevant for anthrax pathogenesis [52], [53]. Our findings provide a biological context where the interaction of TEM8 with LRP6 might be relevant. In the CAM, TEM8 and PA engagement could regulate the availability and function of LRP6/5, favoring beta catenin stabilization. Alternatively, TEM8 and PA could cooperate with Wnt signal transduction pathway converging at regulating either beta catenin phosphorylation, nuclear translocation, and/or transcriptional activity. Our results predict that TEM8 expression in endothelial cells associated to tumor blood vessels would increase the response of these cells to local Wnt production by increasing the intracellular pools of beta catenin available for signaling. Wnt activation in tumor endothelial cells would confer similar growth and invasive properties as in tumor cells.

## Supporting Information

Figure S1 **PA increases activated beta catenin levels in TEM8 expressing HMEC.** Activated Beta Catenin, Beta catenin and tubulin levels detected by western blot in 5 µg of lysate prepared from control or TEM8-recombinant adenovirus infected HMEC treated with 100 ng/ml mWnt3a or 1 µg/ml PA for 3 h. One of 2 experiments yielding similar results is shown.
(TIF)

## References

[1] Niemisto A, Dunmire V, Yli-Harja O, Zhang W, Shmulevich I (2005). Robust quantification of in vitro angiogenesis through image analysis.. IEEE Trans Med Imaging.

[2] Veeman MT, Slusarski DC, Kaykas A, Louie SH, Moon RT (2003). Zebrafish prickle, a modulator of noncanonical Wnt/Fz signaling, regulates gastrulation movements.. Curr Biol.

[3] St Croix B, Rago C, Velculescu V, Traverso G, Romans KE (2000). Genes expressed in human tumor endothelium.. Science.

[4] Carson-Walter EB, Watkins DN, Nanda A, Vogelstein B, Kinzler KW (2001). Cell surface tumor endothelial markers are conserved in mice and humans.. Cancer Res.

[5] Nanda A, Carson-Walter EB, Seaman S, Barber TD, Stampfl J (2004). TEM8 interacts with the cleaved C5 domain of collagen alpha 3(VI).. Cancer Res.

[6] Liu S, Wang H, Currie BM, Molinolo A, Leung HJ (2008). Matrix metalloproteinase-activated anthrax lethal toxin demonstrates high potency in targeting tumor vasculature.. J Biol Chem.

[7] Fernando S, Fletcher BS (2009). Targeting tumor endothelial marker 8 in the tumor vasculature of colorectal carcinomas in mice.. Cancer Res.

[8] Nanda A, St Croix B (2004). Tumor endothelial markers: new targets for cancer therapy.. Curr Opin Oncol.

[9] Bell SE, Mavila A, Salazar R, Bayless KJ, Kanagala S (2001). Differential gene expression during capillary morphogenesis in 3D collagen matrices: regulated expression of genes involved in basement membrane matrix assembly, cell cycle progression, cellular differentiation and G-protein signaling.. J Cell Sci.

[10] Young JA, Collier RJ (2007). Anthrax toxin: receptor binding, internalization, pore formation, and translocation.. Annu Rev Biochem.

[11] Cui X, Li Y, Li X, Laird MW, Subramanian M (2007). Bacillus anthracis edema and lethal toxin have different hemodynamic effects but function together to worsen shock and outcome in a rat model.. J Infect Dis.

[12] Kuo SR, Willingham MC, Bour SH, Andreas EA, Park SK (2008). Anthrax toxin-induced shock in rats is associated with pulmonary edema and hemorrhage.. Microb Pathog.

[13] Bolcome RE, Sullivan SE, Zeller R, Barker AP, Collier RJ (2008). Anthrax lethal toxin induces cell death-independent permeability in zebrafish vasculature.. Proc Natl Acad Sci U S A.

[14] Bromberg-White JL, Boguslawski E, Duesbery NS (2009). Perturbation of mouse retinal vascular morphogenesis by anthrax lethal toxin.. PLoS One.

[15] Duesbery NS, Resau J, Webb CP, Koochekpour S, Koo HM (2001). Suppression of ras-mediated transformation and inhibition of tumor growth and angiogenesis by anthrax lethal factor, a proteolytic inhibitor of multiple MEK pathways.. Proc Natl Acad Sci U S A.

[16] Hotchkiss KA, Basile CM, Spring SC, Bonuccelli G, Lisanti MP (2005). TEM8 expression stimulates endothelial cell adhesion and migration by regulating cell-matrix interactions on collagen.. Exp Cell Res.

[17] Reeves CV, Dufraine J, Young JA, Kitajewski J (2010). Anthrax toxin receptor 2 is expressed in murine and tumor vasculature and functions in endothelial proliferation and morphogenesis.. Oncogene.

[18] Rogers MS, Christensen KA, Birsner AE, Short SM, Wigelsworth DJ (2007). Mutant anthrax toxin B moiety (protective antigen) inhibits angiogenesis and tumor growth.. Cancer Res.

[19] Rmali KA, Puntis MC, Jiang WG (2005). TEM-8 and tubule formation in endothelial cells, its potential role of its vW/TM domains.. Biochem Biophys Res Commun.

[20] Ribatti D, Nico B, Vacca A, Roncali L, Burri PH (2001). Chorioallantoic membrane capillary bed: a useful target for studying angiogenesis and anti-angiogenesis in vivo.. Anat Rec.

[21] Djonov VG, Galli AB, Burri PH (2000). Intussusceptive arborization contributes to vascular tree formation in the chick chorio-allantoic membrane.. Anat Embryol (Berl).

[22] Gu J, Faundez V, Werner E (2010). Endosomal recycling regulates Anthrax Toxin Receptor 1/Tumor Endothelial Marker 8-dependent cell spreading.. Exp Cell Res.

[23] Brooks PC, Clark RA, Cheresh DA (1994). Requirement of vascular integrin alpha v beta 3 for angiogenesis.. Science.

[24] Kurz H, Fehr J, Nitschke R, Burkhardt H (2008). Pericytes in the mature chorioallantoic membrane capillary plexus contain desmin and alpha-smooth muscle actin: relevance for non-sprouting angiogenesis.. Histochem Cell Biol.

[25] Abrami L, Leppla SH, van der Goot FG (2006). Receptor palmitoylation and ubiquitination regulate anthrax toxin endocytosis.. J Cell Biol.

[26] Dimitropoulou C, Malkusch W, Fait E, Maragoudakis ME, Konerding MA (1998). The vascular architecture of the chick chorioallantoic membrane: sequential quantitative evaluation using corrosion casting.. Angiogenesis.

[27] Wilting J, Christ B, Weich HA (1992). The effects of growth factors on the day 13 chorioallantoic membrane (CAM): a study of VEGF165 and PDGF-BB.. Anat Embryol (Berl).

[28] Wilting J, Christ B, Bokeloh M, Weich HA (1993). In vivo effects of vascular endothelial growth factor on the chicken chorioallantoic membrane.. Cell Tissue Res.

[29] Zerlin M, Julius MA, Kitajewski J (2008). Wnt/Frizzled signaling in angiogenesis.. Angiogenesis.

[30] Franco CA, Liebner S, Gerhardt H (2009). Vascular morphogenesis: a Wnt for every vessel?. Curr Opin Genet Dev.

[31] Dejana E (2010). The role of wnt signaling in physiological and pathological angiogenesis.. Circ Res.

[32] Corada M, Nyqvist D, Orsenigo F, Caprini A, Giampietro C (2010). The Wnt/beta-catenin pathway modulates vascular remodeling and specification by upregulating Dll4/Notch signaling.. Dev Cell.

[33] MacDonald BT, Tamai K, He X (2009). Wnt/beta-catenin signaling: components, mechanisms, and diseases.. Dev Cell.

[34] Wei W, Lu Q, Chaudry GJ, Leppla SH, Cohen SN (2006). The LDL receptor-related protein LRP6 mediates internalization and lethality of anthrax toxin.. Cell.

[35] Abrami L, Kunz B, Deuquet J, Bafico A, Davidson G (2008). Functional interactions between anthrax toxin receptors and the WNT signalling protein LRP6.. Cell Microbiol.

[36] Semenov MV, Tamai K, Brott BK, Kuhl M, Sokol S (2001). Head inducer Dickkopf-1 is a ligand for Wnt coreceptor LRP6.. Curr Biol.

[37] Goodwin AM, Sullivan KM, D'Amore PA (2006). Cultured endothelial cells display endogenous activation of the canonical Wnt signaling pathway and express multiple ligands, receptors, and secreted modulators of Wnt signaling.. Dev Dyn.

[38] Kam Y, Quaranta V (2009). Cadherin-bound beta-catenin feeds into the Wnt pathway upon adherens junctions dissociation: evidence for an intersection between beta-catenin pools.. PLoS One.

[39] Staal FJ, Noort Mv M, Strous GJ, Clevers HC (2002). Wnt signals are transmitted through N-terminally dephosphorylated beta-catenin.. EMBO Rep.

[40] Werner E, Kowalczyk AP, Faundez V (2006). Anthrax toxin receptor 1/tumor endothelium marker 8 mediates cell spreading by coupling extracellular ligands to the actin cytoskeleton.. J Biol Chem.

[41] Barolo S (2006). Transgenic Wnt/TCF pathway reporters: all you need is Lef?. Oncogene.

[42] Ausprunk DH, Knighton DR, Folkman J (1974). Differentiation of vascular endothelium in the chick chorioallantois: a structural and autoradiographic study.. Dev Biol.

[43] Caduff JH, Fischer LC, Burri PH (1986). Scanning electron microscope study of the developing microvasculature in the postnatal rat lung.. Anat Rec.

[44] Djonov V, Andres AC, Ziemiecki A (2001). Vascular remodelling during the normal and malignant life cycle of the mammary gland.. Microsc Res Tech.

[45] Ribatti D, Nico B, Floris C, Mangieri D, Piras F (2005). Microvascular density, vascular endothelial growth factor immunoreactivity in tumor cells, vessel diameter and intussusceptive microvascular growth in primary melanoma.. Oncol Rep.

[46] Patan S, Munn LL, Jain RK (1996). Intussusceptive microvascular growth in a human colon adenocarcinoma xenograft: a novel mechanism of tumor angiogenesis.. Microvasc Res.

[47] Hlushchuk R, Riesterer O, Baum O, Wood J, Gruber G (2008). Tumor recovery by angiogenic switch from sprouting to intussusceptive angiogenesis after treatment with PTK787/ZK222584 or ionizing radiation.. Am J Pathol.

[48] Suchting S, Freitas C, le Noble F, Benedito R, Breant C (2007). The Notch ligand Delta-like 4 negatively regulates endothelial tip cell formation and vessel branching.. Proc Natl Acad Sci U S A.

[49] Hellstrom M, Phng LK, Hofmann JJ, Wallgard E, Coultas L (2007). Dll4 signalling through Notch1 regulates formation of tip cells during angiogenesis.. Nature.

[50] Thurston G, Noguera-Troise I, Yancopoulos GD (2007). The Delta paradox: DLL4 blockade leads to more tumour vessels but less tumour growth.. Nat Rev Cancer.

[51] Maher MT, Mo R, Flozak AS, Peled ON, Gottardi CJ Beta-catenin phosphorylated at serine 45 is spatially uncoupled from beta-catenin phosphorylated in the GSK3 domain: implications for signaling.. PLoS One.

[52] Ryan PL, Young JA (2008). Evidence against a human cell-specific role for LRP6 in anthrax toxin entry.. PLoS One.

[53] Young JJ, Bromberg-White JL, Zylstra C, Church JT, Boguslawski E (2007). LRP5 and LRP6 are not required for protective antigen-mediated internalization or lethality of anthrax lethal toxin.. PLoS Pathog.

