# Supplementary figures and images for: Tumor Endothelial Marker 8 Amplifies Canonical Wnt Signaling in Blood Vessels
Source: PLoS One. 2011 Aug 1;6(8):e22334. doi: 10.1371/journal.pone.0022334 (PMC3148219; doi:10.1371/journal.pone.0022334)

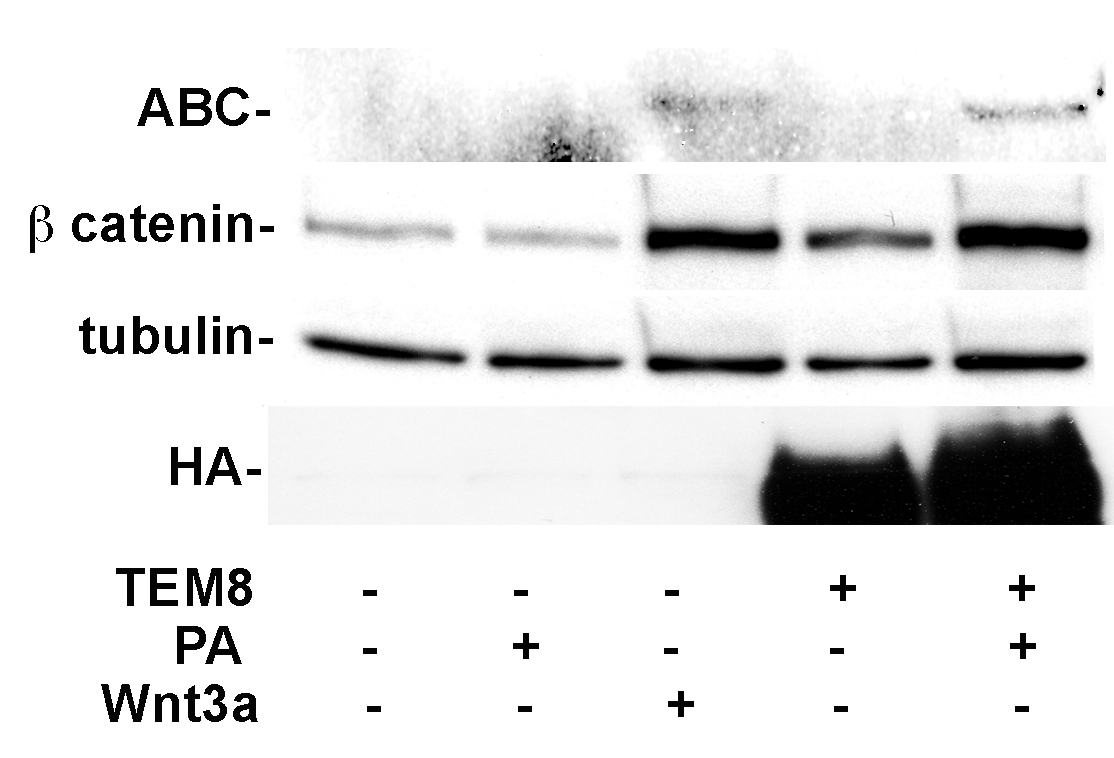

Supplement: Figure S1 — PA increases activated beta catenin levels in TEM8 expressing HMEC. Activated Beta Catenin, Beta catenin and tubulin levels detected by western blot in 5 µg of lysate prepared from control or TEM8-recombinant adenovirus infected HMEC treated with 100 ng/ml mWnt3a or 1 µg/ml PA for 3 h. One of 2 experiments yielding similar results is shown. (TIF) [file pone.0022334.s001.tif]
